# Coordinated transcriptional response to environmental stress by a *Synechococcus* virus

**DOI:** 10.1093/ismejo/wrae032

**Published:** 2024-03-03

**Authors:** Branko Rihtman, Alberto Torcello-Requena, Alevtina Mikhaylina, Richard J Puxty, Martha R J Clokie, Andrew D Millard, David J Scanlan

**Affiliations:** School of Life Sciences, University of Warwick, Gibbet Hill Road, Coventry CV4 7AL, United Kingdom; School of Life Sciences, University of Warwick, Gibbet Hill Road, Coventry CV4 7AL, United Kingdom; School of Life Sciences, University of Warwick, Gibbet Hill Road, Coventry CV4 7AL, United Kingdom; School of Life Sciences, University of Warwick, Gibbet Hill Road, Coventry CV4 7AL, United Kingdom; Leicester Centre for Phage Research, Department of Genetics and Genome Biology, University of Leicester, University Road, Leicester LE1 7RH, United Kingdom; Leicester Centre for Phage Research, Department of Genetics and Genome Biology, University of Leicester, University Road, Leicester LE1 7RH, United Kingdom; School of Life Sciences, University of Warwick, Gibbet Hill Road, Coventry CV4 7AL, United Kingdom

**Keywords:** bacteriophage, cyanobacteria, Synechococcus, nutrient limitation, phosphorus

## Abstract

Viruses are a major control on populations of microbes. Often, their virulence is examined in controlled laboratory conditions. Yet, in nature, environmental conditions lead to changes in host physiology and fitness that may impart both costs and benefits on viral success. Phosphorus (P) is a major abiotic control on the marine cyanobacterium *Synechococcus*. Some viruses infecting *Synechococcus* have acquired, from their host, a gene encoding a P substrate binding protein (PstS), thought to improve virus replication under phosphate starvation. Yet, *pstS* is uncommon among cyanobacterial viruses. Thus, we asked how infections with viruses lacking PstS are affected by P scarcity. We show that the production of infectious virus particles of such viruses is reduced in low P conditions. However, this reduction in progeny is not caused by impaired phage genome replication, thought to be a major sink for cellular phosphate. Instead, transcriptomic analysis showed that under low P conditions, a PstS-lacking cyanophage increased the expression of a specific gene set that included *mazG*, *hli2*, and *gp43* encoding a pyrophosphatase, a high-light inducible protein and DNA polymerase, respectively. Moreover, several of the upregulated genes were controlled by the host’s *phoBR* two-component system. We hypothesize that recycling and polymerization of nucleotides liberates free phosphate and thus allows viral morphogenesis, albeit at lower rates than when phosphate is replete or when phages encode *pstS*. Altogether, our data show how phage genomes, lacking obvious P-stress–related genes, have evolved to exploit their host’s environmental sensing mechanisms to coordinate their own gene expression in response to resource limitation.

## Introduction

Viruses encounter their hosts under a range of environmental conditions. Classically, infection under sub-optimal conditions leads to lysogeny in temperate phages that have a choice between lytic and lysogenic life cycles [1, 2]. Additional studies have shown that the decision-making process leading to a lysogenic lifestyle is not necessarily as straightforward as previously thought and can be the result of other environmental triggers than low nutrient levels, such as high microbial density [3]. In contrast, obligately lytic phages have no choice but to pursue a lytic cycle once they infect their host. While much of phage biology is based on laboratory experimentation under defined nutrient conditions, albeit usually using rich media, relatively little is known of phage infection dynamics under conditions of host nutrient limitation.

For many environmental bacteria, such as cyanobacteria, growth under nutrient-limiting conditions is the norm, especially those marine genera that occupy the vast oligotrophic ocean gyres that are particularly impoverished for the macronutrients N and P [4]. While many of these marine cyanobacteria have optimized their physiology, biochemistry, and genomic footprint (see [5, 6]) to optimize growth under such harsh surroundings, infection by a phage adds yet another burden on the cell. However, compared with host nutrient stress responses, relatively little is known about how such phage–host interactions play out under such sub-optimal growth conditions for the host, particularly the molecular mechanisms that allow phages to lyse such a “sick” host.

Previous work on bacteriophage T4, infecting *Escherichia coli* cells in stationary phase, has shown that bacteriophages infecting cells deprived of nutrients can employ different strategies to deal with this deprivation, in this case altering life strategies from a regular infectious mode to one of “hibernation,” where some phage proteins are produced yet there is no complete synthesis of mature virions until nutrients become available again [7]. Another infection strategy is scavenging of the few nutrients available, together with the utilization of host cellular building blocks, which often get degraded in the course of infection [8, 9], and the production of greatly reduced viral progeny from starved cells [7, 10].

Similar to this *E. coli* work, cyanophage S-PM2d infection of the model marine cyanobacterium *Synechococcus* sp. WH7803 during growth under phosphate-deplete conditions also showed evidence of perturbed infection dynamics, noticeably an extended latent period [11]. In contrast, no significant difference in the timing of cell lysis was observed with cyanophage P-SSM2 infecting the marine *Prochlorococcus* strain NATL2A [12] and cyanophage S-SM1 infecting *Synechococcus* sp. WH8102 [13], following P-deplete host growth. It was suggested that the expression of a gene encoding a phage version of the high affinity phosphate periplasmic binding protein PstS was a key factor here, potentially maintaining phosphate uptake at a time when host gene expression was being shut down [12]. Indeed, expression of this P-related auxiliary metabolic gene (AMG) appears to be controlled by the host two-component PhoBR system, comprising a sensor (PhoR) and a DNA binding response regulator (PhoB), see [14] for a review, suggesting that phages can even hijack host regulatory networks in order to selectively overexpress specific AMGs under the relevant nutrient conditions [12, 13].

Not all phages infecting marine cyanobacteria, which thrive in nutrient-deplete environments, possess obvious orthologues of these host metabolic genes, and, given that such types of sub-optimal infections likely play out in great number in real-world environments, such as ocean gyres where nutrient impoverished sub-optimal host growth occurs, it is of general interest to investigate how obligately lytic phages optimize these infections under nutrient limiting conditions.

In this study, we used a cyanophage-*Synechococcus* model system to specifically address this question, focusing on a phage that lacks obvious AMGs that could potentially help it optimize the infection process under P-deplete conditions. Using whole-cell transcriptomics and electrophoretic mobility shift assays, we describe a new network of genes up-regulated in a cyanophage genome during infection of a P-deplete host, but controlled by the host PhoBR system, that are widely distributed amongst cyanophages and potentially give a broad indication of how a variety of cyanophages undergo a lytic infection under sub-optimal host growth conditions.

## Materials and methods

### Culture growth conditions

*Synechococcus* sp. WH7803 (https://roscoff-culture-collection.org/rcc-strain-details/752) cultures were grown in defined artificial seawater (ASW) medium [11], containing either 172-μM K_2_HPO_4_ for phosphate-replete (ASW + P) growth or transferred at a 1:10 volume into ASW medium lacking phosphate (ASW-P), see below, under continuous illumination at a light intensity of ~10-μmol photons m^−2^ s^−1^ with constant shaking at ~220 rpm. Cyanophage S-PM2d [15, 16] was propagated on *Synechococcus* sp. WH7803 grown in ASW + P medium. Upon lysis, the lysed culture was filtered through a 0.22-μm pore size vacuum filter (Corning, Corning, NY, USA) and the lysate concentrated with 10% w/v PEG 8000. Concentrated phages were resuspended in ASW + P or ASW-P, the PEG removed by centrifugation with chloroform and phages further concentrated in the appropriate medium, using Amicon filter columns (50 000 kDa MW cut-off, Merck, UK). The phage titre was measured via a plaque assay method [17]. Infection parameters were measured either using plaque assays or via one-step growth experiments performed in 96-well plates. To elicit phosphate-deplete infection dynamics daily infection assays were performed using 96-well plates as follows: *Synechococcus* sp. WH7803 (100 ml) was grown in ASW + P medium to an OD_750_ = 0.35. The culture was then transferred into 1 L of fresh ASW + P or ASW-P medium. These freshly transferred cultures were grown under the same conditions as the initial starter culture until reaching an OD_750_ = 0.25. Then, infection assays were performed daily, with *Synechococcus* cell numbers being enumerated by flow cytometry using a FACScan (Becton Dickinson, Franklin, NJ, USA). Each sample count was normalized to counts of multifluorescent beads (Polysciences, Warrington, PA, USA), the concentration of which was prior estimated via fluorescent microscopy. Red and orange fluorescence, accounting for chlorophyll/allophycocyanin and phycoerythrin, respectively, were measured through FL3 (650 nm) and FL2 (585/42 nm) filters, respectively. Cell and bead counts were collected using CellQuest software (Becton Dickinson, UK). P-replete and P-deplete *Synechococcus* sp. WH7803 cells (200 μl) were aliquoted into a 96-well plate in triplicate and cyanophage S-PM2d of known titre added to give a multiplicity of infection (MOI) of 10. A no virus control was also performed, adding ASW or ASW-P medium (100 μl) instead of phage, to wells containing the uninfected host grown under either P-replete or P-deplete conditions. The microtitre plate was then incubated under continuous illumination at an intensity of ~10-μmol photons m^−2^ s^−1^ and the OD_750_ measured every 2 h using an iMark Microplate reader (Bio-Rad, UK). A delayed infection phenotype was indicated when a lysis delay of more than 2 h in the P-deplete, compared with the P-replete culture, was observed. When cells were shown to be sufficiently P-stressed to exhibit a delayed infection, a large-scale infection experiment was performed, and samples were taken every 3 h for qPCR and RNAseq experiments (see below).

### PCR assays to assess the presence of *pstS* in phage genomes

The presence of *pstS* in phage isolates was assessed via PCR amplification using degenerate primers (see Supplementary Table 1). Degenerate primers were designed using the HYDEN program [18]. As an input for the HYDEN script, sequences of putative *pstS* genes from six sequenced cyanophages were used (see Supplementary Table 2). PCR amplifications were performed using MyTaq Mix (Meridian, Memphis, TN, USA) according to the manufacturer’s instructions, over 35 amplification cycles with an annealing temperature of 55°C and elongation time of 30 s.

### qPCR assays

Upon onset of the lysis delay, as detected by the 96-well plate assay described above, *Synechococcus* sp. WH7803 cells growing in either P-replete or P-depleted ASW medium were enumerated using flow cytometry, as described above, and cultures diluted to a final concentration of 5 × 10^7^ cells ml^−1^ in ASW/ASW-P medium, respectively. Diluted cultures (5 ml) were aliquoted into polycarbonate tubes and infected with cyanophage S-PM2d at an MOI of 10 in triplicate. Uninfected cultures were used as a control. Samples were taken at time points 0, 2, 4, 6, 8, 10, 12-, 14-, 16-, and 18 h post-infection, and 200 μl of each sample was diluted in 500-μl ASW medium and vacuum filtered through a 0.2-μm pore size polycarbonate Isopore filter (Merck Millipore, Burlington, MA, USA), mounted on a glass filter tower. While still on the filter, samples were washed three times with 1-ml preservation solution (100-mM EDTA, 500-mM NaCl, 10-mM Tris–HCl, pH 8.0). The filters were then inserted into a ribolyser Lysing Matrix E tube (MP Bioproducts, Irvine, CA, USA) and snap-frozen in liquid nitrogen. An additional 100-μl infected culture was fixed with paraformaldehyde (1% w/v final concentration) and stored at 4°C for subsequent cell counting by flow cytometry.

To obtain cell lysates, 650-μl Tris–HCl pH 8.0 was added to each filter-containing ribolyser tube and cells lysed via 3 cycles of 30 s shaking at 30 Hz in a TissueLyser Qiagen (Retsch GmbH, Germany). The tubes were then subjected to centrifugation at 10 000 g and the supernatant snap-frozen in liquid nitrogen and stored at −80°C. To calculate the intracellular phage DNA concentration, samples were diluted 1:10 (v/v) and qPCR performed as described elsewhere [19].

### Whole-cell transcriptomics

A 1-l culture of *Synechococcus* sp. WH7803 was grown under P-replete and P-deplete conditions, until a delayed lysis phenotype was observed in the P-deplete compared with the P-replete culture (as detected via the 96-well plate assay, see above). Cells were then enumerated using flow cytometry, as described above, and cultures diluted to 5 × 10^7^ cells ml^−1^ in ASW + P/ASW-P medium, respectively. The ASW + P and ASW-P cultures were then divided into six replicates of 100 ml each. Three replicates of each condition were infected with cyanophage S-PM2d at an MOI of 8, while the remaining three replicates were left uninfected as the no-phage control. At each time point, 15-ml sample was taken from each replicate and filtered through a 0.2-μm pore size polycarbonate filter. Filters were washed three times using preservation buffer (100-mM EDTA, 500-mM NaCl, 10-mM Tris–HCl, pH 8.0), snap frozen in ribolyser tubes in liquid nitrogen, and stored at −80°C.

RNA was extracted and purified according to a previous publication [20]. The RNA concentration was quantified with a Qubit Fluorometer (Invitrogen, Waltham, MA, USA) using the Qubit RNA HS Assay and RNA integrity verified using Bioanalyzer RNA 6000 Pico chips (Agilent, Santa Clara, CA, USA).

Samples for RNA sequencing were sent to the Next Generation Sequencing Facility at the Leeds Institute of Biomedical and Clinical Sciences, St James University Hospital, University of Leeds. Libraries were prepared using the ScriptSeq Complete Kit (Bacteria) and ScriptSeq v2 RNA-seq Kit (Illumina, San Diego, CA, USA). Ribosomal RNA was removed using the RiboZero Kit – Bacteria (Illumina). Paired end sequencing was performed on the HiSeq 3000 platform, producing 150 base reads.

Paired reads were mapped to both the cyanophage S-PM2d and *Synechococcus* sp. WH7803 genomes using the BWA MEM v0.7.13 [21] with default parameters. The resulting SAM files were converted to BAM files and sorted using SAMtools v1.3.1 [22]. To count the reads mapping to specific S-PM2d loci, sorted BAM files were first converted to BED files using the Bedtools bamtobed script [23]. BED files were then used to count the reads mapping to different S-PM2d genes using the Bedtools intersect script with “-c -bed -s” options. Reads were normalized using the RPKM model, giving an estimate of relative gene expression calculated according to the following equation:


$$ \mathrm{RPKM}=\frac{\mathrm{Read}\ \mathrm{Count}}{\mathrm{Gene}\ \mathrm{length}\ast{10}^{-3}\ast \mathrm{Total}\ \mathrm{Reads}\ast{10}^{-6}}, $$

where Read Count represents the number of reads mapping to a specific locus, Gene Length is the length of that locus, and Total Reads represents the number of total reads out of each sample mapping to the S-PM2d and *Synechococcus* sp. WH7803 genomes.

To establish changes in gene expression of S-PM2d genes between the corresponding time points in ASW + P and ASW-P infected cultures, differential gene expression values were calculated using the DESeq2 R package [24] in the R Studio environment. The gene expression fold change values, as well as statistical significance, were calculated using the DESeqDataSetFromHTSeqCount function from the DESeq2 package. Only genes with a False Discover Rate (FDR) *P-*value <.05 were considered to be differentially expressed between the conditions.

### Bioinformatics analysis of putative pho boxes in differentially regulated S-PM2d genes

Promoters of S-PM2d genes identified as differentially expressed under −P conditions were examined for the presence of putative PhoB-binding motifs. Promoter sequences were analyzed using the Pattern Locator script [25]. The consensus sequence previously identified as a putative Pho box binding site in cyanobacteria - 5′- PyTTAAPyPyT/A-3′ - [26] was used to scan the upstream region of these S-PM2d genes and several potential binding sites were identified.

### Over-expression of the *Synechococcus* sp. WH7803 PhoB protein in *E. coli*

The *Synechococcus* sp. WH7803 *phoB* gene (Syn_WH7803_01545) was codon optimized for expression in *E. coli* and cloned into the pMAL c4X expression vector (Genscript, Piscataway, NJ, USA) to produce a maltose binding protein (MBP)-PhoB fusion protein. The construct was used to chemically transform NEBExpress competent *E. coli* and transformants selected using 100-μg ml^−1^ ampicillin. After reaching OD_600_ ≈ 0.5 in LB medium, PhoB protein expression was induced using 0.3-mM IPTG for 4 h. Cells were then harvested by centrifugation, resuspended in BugBuster lysis buffer (Merck Millipore), and lysed using a French Press. Soluble and insoluble fractions were separated via centrifugation and the overexpressed MBP-PhoB fusion protein purified via an AKTA fast protein liquid chromatography (FPLC) system (GE Healthcare, Chicago, IL, USA) using an MBPTrap column (GE Healthcare). The amount of purified MBP-PhoB was quantified using a Bradford assay (Thermofisher, Waltham, MA, USA) according to the manufacturer’s instructions. To purify the MBP protein alone, which was used as a control in Electrophoretic Mobility Shift Assays (EMSA), over-expressed MBP-PhoB fusion protein was eluted with 10-mM maltose in loading buffer (20-mM Tris, pH 8; 2-mM MgCl_2_, 200-mM NaCl) and Factor Xa protease (NEB, USA) added to a final concentration of 10 μg ml^−1^ in loading buffer containing 2-mM CaCl_2_ and incubated at 4°C overnight on a shaker. Factor Xa protease was removed using a HiTrap Q XL column (Cytiva, UK) washed with loading buffer. Separated MBP and PhoB proteins were eluted using a 0–1 M NaCl gradient. Purified MBP eluted at ~300 mM NaCl and PhoB at ~500-mM NaCl. Fractions were collected, concentrated, and further purified by size-exclusion chromatography in loading buffer using a Superdex 200 10/300 column (Cytiva, UK) and an AKTA FPLC system (GE Healthcare, Chicago, IL, USA). Note that purified PhoB alone was insoluble as evidenced by the formation of a precipitate in solution after removal of the MBP tag, elution of the protein as an unexpectedly high molecular weight peak on the AKTA column and protein stuck in the well in native PAGE.

### Electrophoretic mobility shift assays

Varying amounts of purified *Synechococcus* sp. WH7803 MBP-PhoB fusion protein were incubated with 25-ng DNA fragments encoding phage gene promoter sequences (see Supplementary Table 1) in 10 μl 2× binding buffer (10-mM Tris–HCl pH 8, 50-mM KCl, 2-mM MgCl_2_, 5% (v/v) glycerol, 0.05-mg mL^−1^ BSA, 1-mM DTT, 3-mM spermidine) for 30 min at 23°C, and 2 μl 5× loading buffer (binding buffer, 20% (v/v) glycerol) was added and the binding reaction run on a pre-cast 10% acrylamide gel (Invitrogen) at 100 V for 90 min at 4°C. The gel was visualized using an ImageQuant LAS 4000 (GE Healthcare). For binding competition assays, DNA fragments were labelled by amplifying the specific genomic region using FAM-labelled primers (IDT, USA) targeting the upstream regions of S-PM2d134 and S-PM2d004 (Supplementary Table 1).

### Genome sequencing of cyanophages S-BM1 and S-BM3

An exponentially growing *Synechococcus* sp. WH7803 culture (100 ml) was infected with 100 μl of filtered cyanophage (S-BM1 or S-BM3) lysate and incubated for several days at 23°C, under illumination at a light intensity of ~10 μmol photons m^−2^ s^−1^ with constant shaking at ~220 rpm. Lysates were subsequently centrifuged at 13 000 g to remove cell debris and filtered through 0.22-μm pore size syringe filters. Filtered lysates were then used to extract viral DNA, using a phenol-chloroform extraction method described in [27].

Purified DNA was then used in Nextera library preparations and sequenced using a MiSeq (Illumina, read length 150 bp) by MicrobesNG (https://microbesng.com/). Fastq files were trimmed with Sickle v1.33, using default parameters [28], and assembled with SPAdes v3.12.0, with the careful option [29]. In each case, the assembly produced a single phage contig with >100-fold coverage. Single contigs where then annotated with Prokka v.1.12 [30] against a custom database [31, 32] derived from previously published bacteriophage genomes.

### Phylogenetic analysis

Cyanophage and cyanobacterial PstS sequences were extracted from the NCBI nr database, Cyanorak (http://abims.sb-roscoff.fr/cyanorak/), and Cyanobase (http://genome.microbedb.jp/cyanobase/), using BLAST. Sequences were aligned using ClustalO [33]. Trees were produced using FastTree 2.1 [34] with default settings, under the Jones–Taylor–Thorton ML model, visualized, and annotated using the iTol phylogenetic trees online annotation tool [35]. *Kyanoviridae* PstS protein sequences were recovered using a uBLAST search against all published *Kyanoviridae* phage genome sequences [31], using the uSEARCH v11 script with the following parameters “-ublast -evalue 1e-20” [36]. Sequences resulting from this query were added to the list of orthologous proteins, aligned, ordered in a phylogenetic tree, visualized, and annotated as described above. To examine congruency between the *Kyanoviridae* cyanophage core tree and the resulting PstS tree, pairwise leaf-leaf distances were extracted from each tree. Weighted and un-weighted Robinson Foulds distances [37] were calculated using TreeDist in R.

The *Kyanoviridae* cyanophage core genome tree was generated using a list of 57 core genes (Supplementary Table 5), as defined in [38, 39]. Hidden Markov Model (hmm) files of each of the core genes were generated using HMMER 3.3.2, with hmmbuild with default settings, followed by hmmpress. Thus, generated hmm files were used to query a database of curated cyanophage genomes [31], and MAFTT v7.490 [40] was used to create separate alignments of each core gene homologue using a custom Perl script listed in Supplementary Data 2. The alignments were concatenated using catfasta2phyml.pl (https://github.com/nylander/catfasta2phyml), and a phylogenetic tree generated using FastTree 2.1 [34] with default settings, under the Jones–Taylor–Thorton ML model, visualized and annotated with the iTol phylogenetic trees online annotation tool [35]. The cyanophage DNA polymerase tree was produced by performing a protein BLAST similarity search against a custom database containing protein sequences of cyanophage genomes using an e-value threshold of 1E−7. The list of 466 cyanophage genomes was generated by searching the INPHARED database [31] for words “cyano,” “Prochlorococcus,” “Synechococcus,” and the names of cyanobacterial genera listed in Supplementary Table 2 of a previous publication [41]. The sequences were aligned and the tree was produced in a manner described above.

A random representative list of cyanophages belonging to the *Kyanoviridae* (previously *Myoviridae)* and *Autographiviridae* (previously *Podoviridae)* families (roughly corresponding to myovirus and podovirus morphotypes, respectively) was generated, covering different clades of the DNA polymerase tree shown in Supplementary Fig. 7 (chosen genomes are marked by a star symbol). For each of those genomes, a Genbank file was generated, containing the DNA polymerase gene and 5 genes upstream and downstream from it, using a custom-made python script listed in Supplementary Data 2. Those GenBank files were used to demonstrate synteny between gene neighbours of the DNA polymerase genes, using Clinker [42].

## Results

### Evolution of PstS within cultured *Kyanoviridae* cyanophages

**Figure 1:**
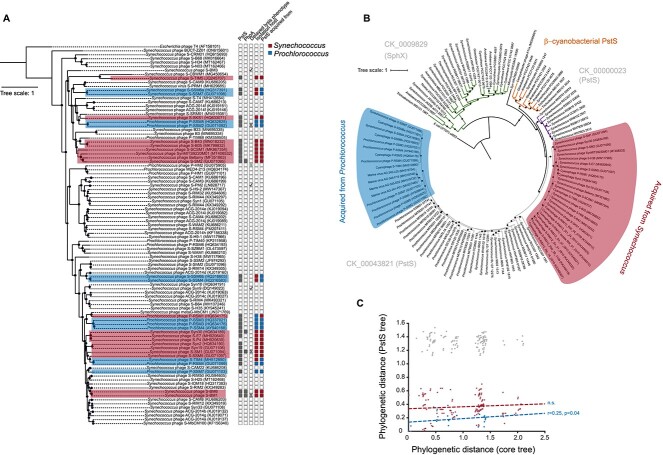
The distribution and evolution of phosphate acquisition AMGs in *Kyanoviridae* cyanophages. (A) Core phylogenetic tree of *Kyanoviridae* cyanophages constructed from 57 gene markers. The tree is rooted on *E. coli* phage T4. The host genus, the presence/absence of *pstS*, *phoA*, and the delayed lysis phenotype is shown for each taxon. Circles on branch junctions indicate bootstrap values >80% (B). Phylogeny of PstS proteins from cyanophages and cyanobacteria. The tree is rooted with *E. coli* PstS. Black circles on branch junctions indicate bootstrap values >80%. (C) Comparison of the phylogenetic distance of cyanophage PstS proteins and their phylogenetic distance in the core tree. Grey dots indicate correlation between all cyanophage PstS proteins, while red and blue signify correlations within the red and blue groups highlighted in (B). Output linear regression statistical tests (Pearson’s correlation) are shown next to the line.

To understand the distribution and evolution of the *pstS* gene in cyanophages, we analyzed 95 currently available complete genomes from the *Kyanoviridae* cyanophages. Of these, *pstS* was present in 30 isolates (~1/3). Genomes containing *pstS* were distributed across the entire phylogeny of this group (Fig. 1A), suggesting either widespread loss or frequent acquisition of the gene. To understand which, we constructed a phylogenetic tree of cyanophage PstS proteins as well as freshwater and marine host proteins (Fig. 1B). Unlike *E. coli*, *pstS* in cyanobacteria is often found in multi-copy (see [43]). Here, we utilize the Cyanorak database [44] to assign a *sphX* cluster (CK_00001829) and two *pstS* clusters (CK_00000023 and CK_00043821), while taxonomically we also identify a discrete β-cyanobacterial, “freshwater-dominated” PstS cluster which encompasses both the *pstS1* and *pstS2* genes characterized experimentally by [43]. Cyanophages possess only *pstS* cluster CK_00043821. The absence of *sphX* or *pstS* cluster CK_00000023 in cyanophages is notable, given that *Synechococcus* hosts frequently possess all three types (themselves often in multiple copy), whereas *Prochlorococcus* hosts only possess the *pstS* CK_00043821 cluster. The freshwater PstS cluster shown in Fig. 1B encompasses proteins encoded by the *pstS1* and *pstS2* genes in *Synechocystis* sp. PCC6803 which are known to have quite different transport kinetics for inorganic phosphate [43]. Thus, it is difficult to predict functional properties of PstS proteins from sequence alone, which precludes understanding why cyanophages have only co-opted the CK_00043821 PstS cluster.

Our phylogeny suggests that *pstS* has been acquired twice in marine cyanophages, once from *Synechococcus* and once from *Prochlorococcus* (Fig. 1B). In most cases (24/30), the donor genus (*Synechococcus* or *Prochlorococcus*) corresponds to the isolation host of the phage, even though isolation host is not phylogenetically conserved (Fig. 1A). Thus, it is likely that these PstS variants facilitate specific interaction with the cognate PstABC membrane bound components of the transport system of their preferred host (see also Zhao *et al.* [45]). In the six cases where *pstS* has not been acquired from the host genus, it is not clear what the host ranges of these phages are, and therefore, the interaction of these “incorrect” PstS types with their host’s PstABC requires further investigation. Within each acquisition event, we assessed the congruency between PstS subtrees and the core phylogeny. For those *pstS* genes acquired from *Prochlorococcus*, there was a significant but weak correlation between phylogenetic tree topologies. For *Synechococcus*, there was no significant correlation (Fig. 1C), suggesting that once acquired, there is frequent horizontal gene transfer of *pstS* between phages. This may explain their rather patchy distribution across the core phylogenetic tree (Fig. 1A). The distribution of *pstS* in these phage genomes could reflect niche adaptation in low P environments, as has been observed in marine metaviromes [46-48]. Similar to previous reports [39], we observed that the *phoA* gene encoding a putative alkaline phosphatase is only present in phage genomes that also possess a *pstS* gene (Fig. 1A).

### PstS-lacking cyanophages show delayed lysis in response to low P

**Figure 2:**
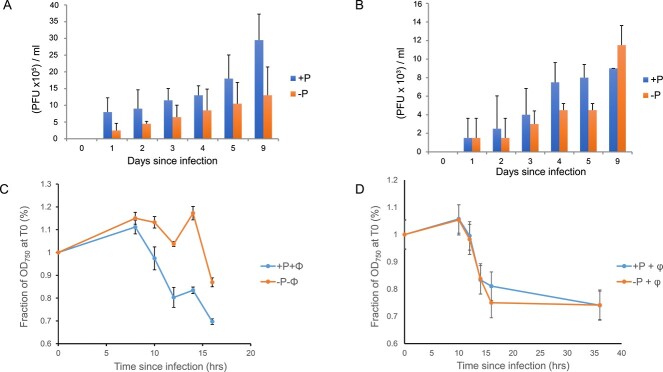
Plaque assay results (A) and (B) and OD_750_ values (C) and (D) of cyanophage infection of *Synechococcus* sp. WH7803 grown under P-replete and P-deplete conditions. Cyanophage used: (A) and (C) S-PM2d; (B) and (D) S-BM1. Error bars represent the standard error of the average of three replicates. T-tests between the number of plaques at the last time point show statistically significant differences between +P and −P-infected samples, both for S-PM2 (*t-*value: 3.511, *P-*value: .025) and S-BM1 (*t*-value: −3.024, *P*-value: .039).

To assess whether the absence of *pstS* in cyanophage genomes more generally correlated with a delayed lysis phenotype [11], we tested PstS-lacking cyanophages isolated from a variety of marine environments under +P/−P conditions (Supplementary Table 3). We used both plaque assays (Fig. 2A and B), measuring the yield and the potential delay in production of plaques, as well as one-step infection assays, measuring the OD_750_ of phage-infected cells over the latent period of infection that indicates when cell lysis occurs (Fig. 2C and D). We further used degenerate primers targeting the phage *pstS* (Supplementary Table 1), to assess the presence of *pstS* in genomes of cyanophages isolated from a variety of marine environments (Supplementary Table 3).

We found a strong correlation between the absence of a *pstS* homologue in the phage genome and the presence of the delayed lysis phenotype when infecting under −P conditions. Of the 18 phages tested, 17 lacked *pstS* and showed a severe delay in lysis (see Fig. 2C for one example). In contrast, a newly sequenced phage, S-BM1, which encodes PstS, had identical latent periods under both P deplete and P replete conditions (Supplementary Table 3, Fig. 2D) in agreement with previous data for two other *pstS*-possessing cyanophages P-SSM2 and S-SM1 that also show no delayed lysis [12, 13]. Therefore, it appears that cyanophage-encoded PstS supports the phosphorus demand of an infected cell to avoid delayed lysis.

### Is the DNA replication rate affected in *pstS*-lacking cyanophage infecting a P-deplete host?

**Figure 3:**
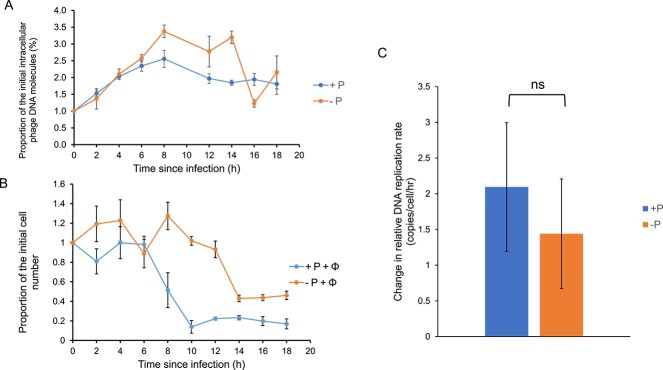
DNA replication rate of cyanophage S-PM2d during infection of *Synechococcus* sp. WH7803 under P-replete and P-deplete conditions. (A) Percentage of the initial amount of intracellular S-PM2d phage DNA over the course of infection under P-replete and P-deplete conditions. (B) The percentage of initial *Synechococcus* sp. WH7803 cell abundance following infection with cyanophage S-PM2d under P-replete and P-deplete conditions. (C) The relative cyanophage DNA replication rate under P-replete and P-deplete conditions. The rate was estimated by calculating the slope of a curve representing the change in the amount of DNA per hour for each of the three replicates, between 2- and 6-hour time points post infection. Error bars represent the standard deviation of three replicates.

Since phage PstS likely controls proper lysis time in response to P availability, we turned our attention to phages lacking *pstS*, in a bid to understand how they were limited. We hypothesized that phages lacking *pstS* would have a decreased DNA replication rate when infecting a P-deplete host. However, for the *pstS*-lacking cyanophage S-PM2d, phage DNA replication rates were not statistically different 2–6 h post-infection between the P-replete and P-deplete conditions (2 Sample T-test values: *t* = 0.78, *P* = .48, Fig. 3A and C).

The dramatic impact on phage infection kinetics with a lack of P in the medium on those cyanophages that do not possess *pstS* (Fig. 2A and C, Supplementary Table 3) points toward a distinct molecular mechanism, either controlled by the host or the phage, by which the infection process is delayed, and the burst size reduced, despite still maintaining similar DNA replication rates (Fig. 3A and C). From the host perspective, this mechanism would reduce the rate of infection of the P-depleted host by the phosphorus-demanding phages, allowing the persistence of the host population throughout periods of P-scarcity in the dynamic marine environment. From the cyanophage perspective, the altered kinetics would mean an alternative to undertaking an abortive infection pathway, and even though the infection is significantly affected and sub-optimal, the low number of viral progeny produced would still allow phage persistence, facilitating future host infection once the depleted nutrient(s), in this case P, become available again.

How then, does the cyanophage maintain a DNA replication rate similar to nutrient replete conditions when infecting a P-deplete grown host? Or how does the phage (or indeed host) control the latent period or burst size? To determine this, we undertook a transcriptomics approach comparing gene expression under P-replete/P-deplete host growth.

### Differential gene expression in response to P availability

*Synechococcus* sp. WH7803 grown in either P-replete or P-deplete conditions was infected with cyanophage S-PM2d at an MOI of 8, and samples taken for RNA analysis at *t* = 3, 6, and 9 h or additionally at *t* = 12 and 15 h post-infection under P-deplete host growth. Sequence coverage and mapping statistics of this RNAseq data are shown in Supplementary Table 4. We noted that the relative abundance of transcripts mapping to the *Synechococcus* host genome in the infected samples is significantly lower, compared with the uninfected control (Supplementary Fig. 1 and Supplementary Table 4), pointing to the immediate degradation of the host transcriptome, similar to what has been observed previously [8, 19]. This reiterates the importance of AMGs in phage genomes, with host transcription being abruptly halted and phage relying only on host genes expressed in the early stages of infection as well as its own genes to facilitate the metabolic needs of its infection cycle.

We did not observe any differentially expressed host genes in response to P conditions in infected cells (data not shown). We expect that this is due to the above-mentioned degradation of host mRNAs early in infection. In contrast, 14 S-PM2d genes were statistically significantly up-regulated 3 h post-infection under P-deplete conditions, compared with the P-replete control (Table 1; Supplementary Fig. 2). These included six S-PM2d genes located in close proximity on the genome, i.e. S-PM2d131 to S-PM2d136 of which S-PM2d133 encodes DNA polymerase (gp43), S-PM2d134—a UvsX RecA-like recombination protein, S-PM2d135—a DNA primase-helicase (gp41), and S-PM2d136—the MazG pyrophosphatase [49]. Most of the other 8 significantly up-regulated genes are annotated as encoding hypothetical proteins apart from S-PM2d118 (gp46), encoding a recombination endonuclease subunit, and S-PM2d172, encoding a high-light inducible protein. There were an additional three genes that were identified as differentially expressed but are probably misannotated in this reference genome (Table 1).

**Table 1:** Differentially expressed cyanophage S-PM2d genes during infection of *Synechococcus* sp. WH7803 under P-deplete compared with P-replete conditions.

**CDS**	**Genbank accession number**	**Product**	**Gene**	**Start codon position**	**Stop codon position**	**Strand**	**log2 Fold Change**	***P-*value**	**FDR-adjusted *P*-value**	**Presence in % of cyanophage genomes**
S-PM2d004	CFW42138.1	Hypothetical protein		1255	1560	+	0.79	3.06E−03	4.36E−02	24.44% (143/585)
S-PM2d006	CFW42141.1	Hypothetical protein		1913	2116	+	1.12	6.28E−04	1.60E−02	2.74% (16/585)
S-PM2d074*^a^	CFW42190.1	Hypothetical protein		14 226	14 432	−	1.03	1.74E−03	3.06E−02	0.34% (2/585)
S-PM2d241	CFW42194.1	Hypothetical protein		14 805	15 005	+	1.00	1.51E−03	2.88E−02	0.17% (1/585)
S-PM2d115	CFW42285.1	Hypothetical protein		75 481	75 699	+	0.63	4.18E−03	5.03E−02	13.50% (79/585)
S-PM2d118	CFW42290.1	Recombination endonuclease subunit	gp46	77 014	78 744	+	0.98	1.61E−04	6.15E−03	74.70% (437/585)
S-PM2d131	CFW42313.1	Hypothetical protein		87 134	87 601	+	1.12	5.76E−05	4.40E−03	9.40% (55/585)
S-PM2d132	CFW42314.1	Hypothetical protein		87 598	87 849	+	1.04	1.18E−04	5.86E−03	9.23% (54/585)
S-PM2d133	CFW42316.1	DNA polymerase	gp43	87 849	90 341	+	0.63	2.62E−03	3.99E−02	84.44% (494/585)
S-PM2d134	CFW42318.1	UvsX RecA-like recombination protein	UvsX RecA-like	90 352	91 383	+	0.84	1.21E−03	2.78E−02	75.04% (439/585)
S-PM2d135	CFW42319.1	DNA primase-helicase	gp41	91 343	92 755	+	0.67	3.24E−03	4.36E−02	74.53% (436/585)
S-PM2d136	CFW42320.1	Pyrophosphatase	mazG	92 757	93 164	+	1.33	1.11E−05	2.11E−03	74.53% (436/585)
S-PM2d169	CFW42374.1	Hypothetical protein		114 418	114 768	+	1.42	1.84E−05	2.11E−03	26.67% (156/585)
S-PM2d172	CFW42378.1	High-light inducible protein	hli2	115 295	115 414	+	0.76	1.41E−03	2.88E−02	50.94% (298/585)
S-PM2d190	CFW42403.1	Hypothetical protein		132 902	133 072	+	0.87	3.10E−04	1.01E−02	0.85% (5/585)
S-PM2d250*^b^	CFW42449.1	Putative ATPase		155 233	155 376	+	0.71	3.19E−02	1.61E−01	0.17% (1/585)
S-PM2d216*^b^	CFW42450.1	Hypothetical protein		155 436	155 594	+	0.91	2.01E−03	3.29E−02	0.17% (1/585)

*Indicates that the CDS is most likely misannotated in the reference genome since ^a^The CDS is on the negative strand and contains no orthologs in cyanophage genomes, whilst there is a more probable overlapping ORF on the positive strand which is shared amongst cyanophages but is not differentially expressed. ^b^These are very small ORFs (<50 aa’s), not shared across cyanophage genomes, and are located within a tRNA operon.

### Several of the differentially regulated S-PM2d genes under P-deplete host growth are controlled by the *Synechococcus* PhoBR system

Since these cyanophage S-PM2d genes were specifically upregulated following the infection of a P-deplete host, we sought to understand how these genes were up-regulated. Given it is known that the *pstS* gene in cyanophage P-SSM2 infecting marine *Prochlorococcus* is controlled by the host two-component system PhoBR [13]. we investigated whether this was the case for these genes as well. Using a Pho box consensus motif previously described for marine *Synechococcus* as comparison [26], bioinformatics and visual inspection of promoter regions showed that 7 of the 14 up-regulated genes possessed a putative Pho box (see Table 2). To experimentally confirm that some of these putative Pho boxes were functional, the *Synechococcus* sp. WH7803 PhoB protein was over-expressed and purified in *E. coli* as a MBP-PhoB fusion protein and the purified protein used in electrophoretic mobility shift assays with DNA fragments of S-PM2d upstream regions either (i) containing a putative Pho box and differentially expressed (genes S-PM2d004, S-PM2d133, S-PM2d136), (ii) containing a putative pho-box, but not differentially expressed (gene S-PM2d130), or (iii) lacking a predicted Pho box but differentially expressed (S-PM2d134).

**Table 2:** The sequence and position of putative Pho boxes found upstream of the differentially expressed cyanophage S-PM2d genes during infection of *Synechococcus* sp. WH7803 under P-deplete compared with P-replete conditions.

**Gene name**	**Gene annotation**	**Putative Pho Box**	**Position**
S-PM2d004	hypothetical protein	**CTTTCCTC**TCG**TTTAACTG ATTATACA**ATT**CTTGGAGA**	−62 and − 25
S-PM2d006	hypothetical protein	NA	
S-PM2d074	hypothetical protein	NA	NA
S-PM2d115	hypothetical protein	NA	NA
S-PM2d118	recombination endonuclease subunit (gp46)	NA	NA
S-PM2d130	Heat shock protein (Hsp20)	**ATTCAAAC**TCG**CTTAAATA**	−31
S-PM2d131	hypothetical protein	**TTTGAAGAT** *GG*T**CTTTTGAA***GA*T**CTCACTGG**	−138
S-PM2d132	hypothetical protein	**CTAATCCA** *AAC* **CTTGATGC**	−91
S-PM2d133	DNA polymerase (gp43)	**GTTAAAAT**ATC**CATATGCA**	−162
S-PM2d134	UvsX RecA-like	NA	
S-PM2d135	DNA primase-helicase (gp41)	NA	NA
S-PM2d136	Pyrophosphatase (m*azG*)	**CTTTTGAA** *GAC* **CTTTCAAC** *TCG* **CCAAAAGC**	−63
S-PM2d169	hypothetical protein	NA	
S-PM2d172	hli2	NA	NA
S-PM2d190	hypothetical protein	NA	NA
S-PM2d216	hypothetical protein	NA	NA
S-PM2d241	hypothetical protein	**CTTCCCCG**C*CG***CTGAGGTG**	−105
S-PM2d250	putative ATPase	**CCTATACT** *GAT* **CTCAGTTC**	−30

Bold nucleotides represent those pertaining to the Pho box, while underlined nucleotides are conserved compared with the recognized consensus Pho box binding site (5'-PyTTAAPyPyT/A-3′) [26]. NA—no putative Pho box was identified. Position is the number of nucleotides upstream of the putative transcriptional start site of that gene.

**Figure 4:**
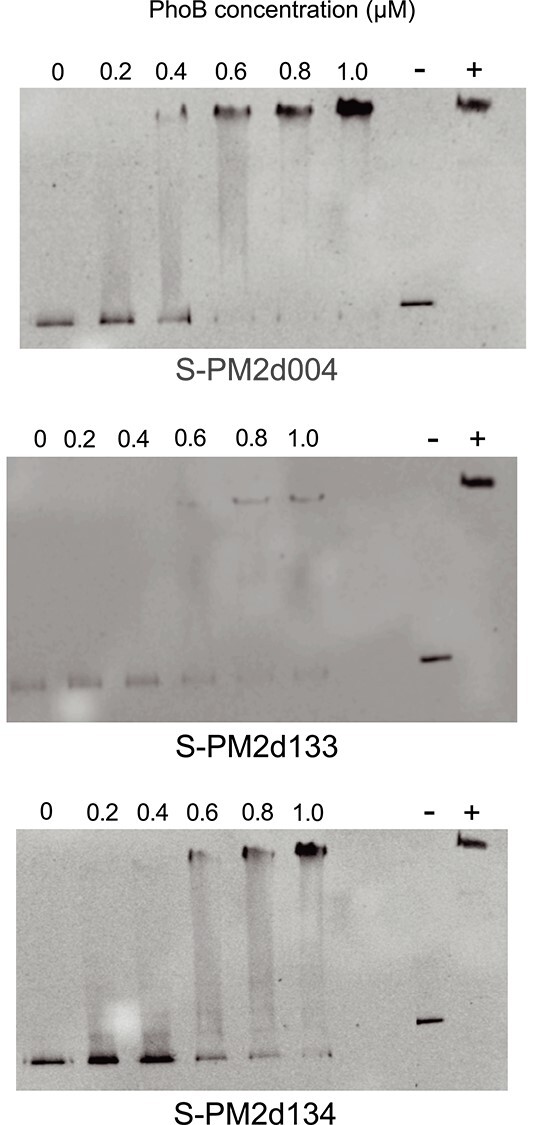
EMSA of purified MBP-PhoB from *Synechococcus* sp. WH7803 with specific cyanophage S-PM2d gene promoters. The concentration of MBP-PhoB protein used ranged from 0 to 1 μM, while 25-ng DNA fragment was used in each case. (−): Negative control, PhoB with an internal fragment of the *Synechococcus* sp. WH7803 *phoB* gene. (+): Positive control, PhoB with the promoter region of the *Synechococcus* sp. WH7803 *phoB* gene.

Of the upstream regions of the genes tested, the MBP-PhoB fusion protein bound to the promoters of S-PM2d004 (encoding a hypothetical protein), S-PM2d133 (encoding DNA polymerase), both with a predicted Pho box but also to SPM2d134 (encoding a putative UvsX, RecA-like protein), which lacks a predicted Pho box (Fig. 4). These three promoters showed no binding to the purified MBP alone (Supplementary Fig. 6). S-PM2d136 showed no binding to the PhoB-MBP fusion (Supplementary Fig. 3), despite being differentially expressed in response to low P and possessing a predicted Pho box. However, this gene may be co-transcribed with the upstream genes S-PM2d135 and S-PM2d134, the latter experimentally determined to bind PhoB. Alternatively, one or more of the over-expressed genes may be controlled by another transcription factor that is in turn under PhoB control. Gene S-PM2d130 had a predicted Pho box but neither bound PhoB (Supplementary Fig. 6) nor was differentially expressed in response to low P. Thus, bioinformatics prediction of Pho boxes in phage genomes is not reliable, and instead experimental validation of PhoB binding better predicts whether the gene was differentially expressed in response to low P. To confirm the specificity of PhoB binding, we performed competition assays in which fluorescently labelled upstream DNA fragments for S-PM2d004 and S-PM2d134 were competed by identical unlabelled DNA fragments, where the binding of the protein to the promoter was outcompeted by increasing the concentration of the unlabelled promoter-containing DNA fragment (Supplementary Fig. 4A), while competition with a non-binding fragment (S-PM2d136) showed no effect (Supplementary Fig. 4B).

In order to confirm that it is indeed the predicted Pho box to which the overexpressed PhoB protein is binding, we progressively reduced the size of these fragments to show that for both the S-PM2d004 (Supplementary Fig. 5A) and S-PM2d133 (Supplementary Fig. 5B) genes only those DNA fragments containing the predicted PhoB binding site resulted in a gel shift.

## Discussion

Cyanophage infection represents a potentially important loss factor for picocyanobacterial populations throughout the global ocean via cell lysis (e.g. see [50]) but also has wide ranging implications for estimates of primary production given their ability to directly inhibit CO_2_ fixation [51]. Thus, given that cyanophage infections can theoretically occur under every environmental condition possible, it is important that we have a mechanistic understanding of how infection dynamics vary as a function of these *in situ* host growth conditions, given this may directly affect the latent period (the time before cell lysis, but also the time during which CO_2_ fixation is inhibited) and burst size (the number of phages produced).

Phosphorus availability has previously been shown to affect cyanophage productivity in picocyanobacteria [12, 13] but with no observed delayed lysis period. Here, we found a more general relationship between the presence of a *pstS* homologue in phage genomes and the lack of the delayed lysis phenotype under −P conditions (Fig. 1A; Supplementary Table 3). The acquisition of *pstS* may therefore enable cyanophages to overcome a bottleneck affecting lysis during phage morphogenesis. Phage *pstS* transcripts can be used to either produce additional copies of the periplasmic P-binding PstS protein, thus increasing the ability to acquire any available phosphate, or they can supplement the mRNA encoding the host *pstS* which is degraded together with the rest of the host transcriptome during the course of infection ([8]; Supplementary Fig. 1), thus providing a continuous supply of the PstS protein in the periplasm of the infected cell. A similar strategy has been shown to be used by cyanophages carrying a photosynthetic gene *psbA*, which encodes a functional D1 protein and enables maximal energy production under high-light conditions in the infected cyanobacterial cell [19, 52, 53].

Phylogenetic examination of the cyanobacterial *pstS* shows strong clustering between the viral genes and the genes of the specific host these viruses infect (Fig. 1B). Thus, *pstS* sequences from *Synechococcus* phages form a sister clade to the *Synechococcus pstS* sequences, while the *Prochlorococcus* viral *pstS* sequences group together with the cyanobacterial *Prochlorococcus pstS* clade. This clear phylogenetic relationship becomes less obvious when looking at the totality of *Kyanoviridae* phages. Here (Fig. 1A), the P-stress–related gene *pstS*, as well as the putative alkaline phosphatase-encoding *phoA*, seem to have evolved numerous times in different phages and do not show clustering that is apparent when looking only at the cyanobacterial and cyanophage *pstS* sequences (Fig. 1B). Additionally, there seems to be no synteny conservation between *pstS* homologs found in different isolated cyanophage genomes, further supporting the multiple origin theory [54]. This observation points to the possibility that adopting host *pstS* is not the only strategy available to phages infecting hosts under P-deplete conditions and that alternative infection strategies, one of which is described in this work, may be more prevalent than we previously thought. The existence of alternative strategies for optimizing infection under different P-conditions, the lack of synteny of *pstS*-surrounding genes between cyanophage isolates, and its apparent sporadic and repeated acquisition over ecological timescales, all suggest that, unlike their cyanobacterial hosts, cyanophages have not evolved into phosphorus-adapted ecotypes.

We have also shown that the delayed lysis phenotype of cyanophage S-PM2d during infection of a P-deplete host [11] appears to be a general phenotype of *pstS* lacking cyanomyoviruses (Supplementary Table 3). Given that S-PM2d genome replication rate was comparable during the infection of a P-replete or P-deplete host (Fig. 3A and C) excludes this as the underlying mechanism. Indeed, we show that under P-deplete conditions, phage genes for DNA replication are specifically upregulated and that phage genomes have evolved to exploit the host’s regulatory system to control this. Since the infectious burst size is 5-fold lower under P-deplete conditions (Fig. 2; [11]), replicated phage genomes do not appear to get incorporated into infectious virions. How and why cyanophages maintain the synthesis of DNA, which is extremely phosphorus demanding (e.g. almost 50% of cellular phosphorus is in DNA [55]), is initially puzzling. However, cyanophages have an abundant source of nucleotides in the form of the host’s chromosome which is immediately degraded upon infection [56], liberating free nucleotides. In *E. coli* phage T4, host chromosome degradation is catalyzed by a combination of the endonuclease II product of the *denA* gene and the gene product of *d2a* [57]. While no homologues of these exist in *Kyanoviridae* cyanophages, it is likely that another endonuclease performs this function. In unrelated cyanophages, host mRNAs are degraded in a process thought be a result of the host’s RNase E, with phages protecting their mRNAs by antisense transcription [8]. A similar mechanism may exist in *Kyanoviridae* cyanophages where antisense transcription is observed [8]. Free NTPs can be converted to dNTPs by the activity of the cyanophage-encoded ribonucleotide reductase [58, 59]. Altogether, it is likely that there is enough free or recycled nucleotides derived from host nucleic acids to meet the demands of phage chromosome replication. Moreover, DNA replication itself releases intracellular phosphate, via the pyrophosphatase activity of DNA polymerase [60]. Since *Synechococcus* cells infected under P-deplete conditions produce 12 new cyanophages on average [11, 61] and that each S-PM2d genome is ~200 kb in length [16], while the addition of each nucleotide to the growing DNA chain produces 2 phosphate molecules, we estimate that during the course of DNA replication, ~10^6^ molecules of phosphate are released by the activity of DNA polymerase alone. Previous work has shown that *Synechococcus* cells growing at external phosphate concentrations of 1 μM accumulate a similar amount of phosphate per cell per hour [62]. Thus, DNA replication may provide a rich source of phosphorus for high P demanding processes of phage morphogenesis.

We also observed that the phage *mazG* (S-PM2d136, Table 1) is up-regulated under P deplete conditions. S-PM2d *mazG* encodes a pyrophosphohydrolase which was initially hypothesized to play a role in suppressing the stringent response in infected cells [63]. However, our previous work showed that the viral MazG does not hydrolyze the alarmone nucleotides ppGpp and pppGpp, but rather hydrolyses nucleotides, with increased affinity for dGTP and dCTP [49]. Since cyanophage S-PM2d has a lower %GC (37.7%; [16]) compared with its *Synechococcus* sp. WH7803 host (60.2%; [64]), it was proposed that the viral MazG has a role in preferentially hydrolysing nucleotides for which it has lower demand, thus providing a potential additional source of intracellular phosphate produced by hydrolysis of less-required nucleotides. This observation further supports our model of amplification of nucleotide metabolism (including DNA replication) processes to provide a novel source of intracellular phosphate. In reality, there may even be an excess of nucleotides for DNA replication. Instead, cyanophages may scavenge phosphate from these to meet other phosphorus demands such as protein synthesis and packaging during the later stages of infection which have been implicated as the main energetic sink for phage production [19]. Thus, it is possible that the role of the phosphate produced intracellularly during phage DNA replication is to provide the energy for the later stages of the latent phase of infection.

Since viral DNA polymerase seems to play an important role in the P-stress response of this group of cyanophages, we examined the phylogeny of DNA polymerases from published genomes of isolated cyanophages (Supplementary Fig. 7), as well as the preservation of synteny of DNA polymerase in these genomes (Supplementary Fig. 8). The phylogeny of DNA polymerase seems to align with the cyanophage family phylogeny with the main clades clustering along the family divide, with the *Kyanoviridae* family members possessing the Type B DNA polymerase, whereas the *Autographiviridae* contain the Type A DNA polymerase (Supplementary Fig. 7). There is no separate clustering between the *Kyanoviridae* DNA polymerases belonging to phages infecting *Prochlorococcus* and *Synechococcus* hosts, pointing toward a possibility of interchangeable acquisition of the gene from the common host of these cyanophages. Noteworthy is the split nature of the DNA polymerase gene found in most of the *Autographiviridae* strains (19/32). In these phages, DNA polymerase is divided into two or three ORFs, found next to each other, often partially overlapping, and encoded in a different reading frame to one another. This feature seems to be conserved among cyanophages from this family, as the second and third polymerase fragments all cluster together in a separate branch within the *Autographiviridae* clade. While this feature has been reported before [65], further work is required to establish whether this curious feature has any biological importance.

For both the *Kyanoviridae* and *Autographviridae* DNA polymerases, there is a high degree of synteny conservation within the two clades. In the case of *Kyanoviridae* polymerases, including the one belonging to cyanophage S-PM2d described in this work, there is the conserved presence of a helicase, UvsX-like recombinase, putative heat-shock protein, and MazG homologues surrounding the DNA polymerase (Supplementary Fig. 8A). Since we find all of these genes, together with the DNA polymerase, to be transcriptionally overexpressed during infection under P-deplete conditions, it is possible that this synteny is conserved as a part of a general cyanophage adaptation to infection under varying P-stress conditions. Certainly, this strategy appears to have been specifically selected for in S-PM2d given DNA polymerase (S-PM2d133) is under direct control of the host P-stress transcriptional regulation response (Fig. 4). Noteworthy here is that the host DNA polymerase is not under the same P-stress control [66, 67]. It is also notable that in the case of some cyanophages infecting both *Synechococcus* and *Prochlorococcus*, the immediate genomic context of the DNA polymerase also contains a *pstS* homologue. This proximity of *pstS* and DNA polymerase in some of these *Kyanoviridae* cyanophage genomes might explain the origin of the Pho-binding motifs in the promoters of these latter genes. The genomes of *Autographiviridae* cyanophages, although also showing a high degree of synteny around their DNA polymerases (Supplementary Fig. 8B), contain a somewhat different (compared with *Kyanoviridae* genomes) list of putative genes located in this genomic neighbourhood: ssDNA binding protein, RNaseH, nucleotide kinase, ribonucleotide kinase and RNA polymerase, with the conservation of *maz*G between these families, a gene which has previously been identified as a part of the core genome of *Kyanoviridae* cyanophages [38, 39].

Overall, our results indicate that bacteriophages not only adapt to nutrient stress by incorporating host metabolic genes into their own genomes [13] but also acquire stress-specific promoters to exploit host transcription factors related to environmental stress. This mechanism presents an exciting new model of viral evolution which should be examined in other virus–host models.

## Supplementary Material

Rihtman_Cyanophage_Supplementary_data_1_wrae032

## Data Availability

The datasets generated during the current study are available as follows: Genome sequences of cyanophages S-BM1 and S-BM3 are available in Genbank under accession numbers OQ319120 and OQ319121, respectively. Paired-end reads used for cyanophage transcriptomics analyzes have been deposited in the European Nucleotide Archive (ENA) at EMBL-EBI under accession number PRJEB64420.
