# Supplementary material for: Coordinated transcriptional response to environmental stress by a Synechococcus virus
Source: ISME J. 2024 Mar 3;18(1):wrae032. doi: 10.1093/ismejo/wrae032 (PMC10976474; doi:10.1093/ismejo/wrae032)
Supplement: Rihtman_Cyanophage_Supplementary_data_1_wrae032 [file rihtman_cyanophage_supplementary_data_1_wrae032.docx]

**Supplementary Information** for:

**Coordinated transcriptional response to environmental stress by a *Synechococcus* virus**

B. Rihtman^1,3^, A. Torcello^1^, A. Mikhaylina^1^, R. J. Puxty^1^, M. R. J. Clokie^2^, A. D. Millard^2^, D. J. Scanlan^1^

^1^ School of Life Sciences, University of Warwick, Gibbet Hill Road, Coventry, UK

^2^ Leicester Centre for Phage Research, Department of Genetics and Genome Biology, University of Leicester, University Road, Leicester, UK

^3^ Corresponding author: b.rihtman.1@warwick.ac.uk

Supplementary Figures

**Supplementary Figure 1:** The percentage of total RNA sequencing reads mapping to the S-PM2d or *Synechococcus* sp. WH7803 genome over the course of the 15-hour infection period. Error bars represent the standard error of three replicates.

**Supplementary Figure 2:** RPKM values of those S-PM2d genes that were found to be differentially expressed at time point 3 hours post infection of *Synechococcus* sp. WH7803 under P-deplete compared to P-replete conditions over the course of the 15 hour infection period.


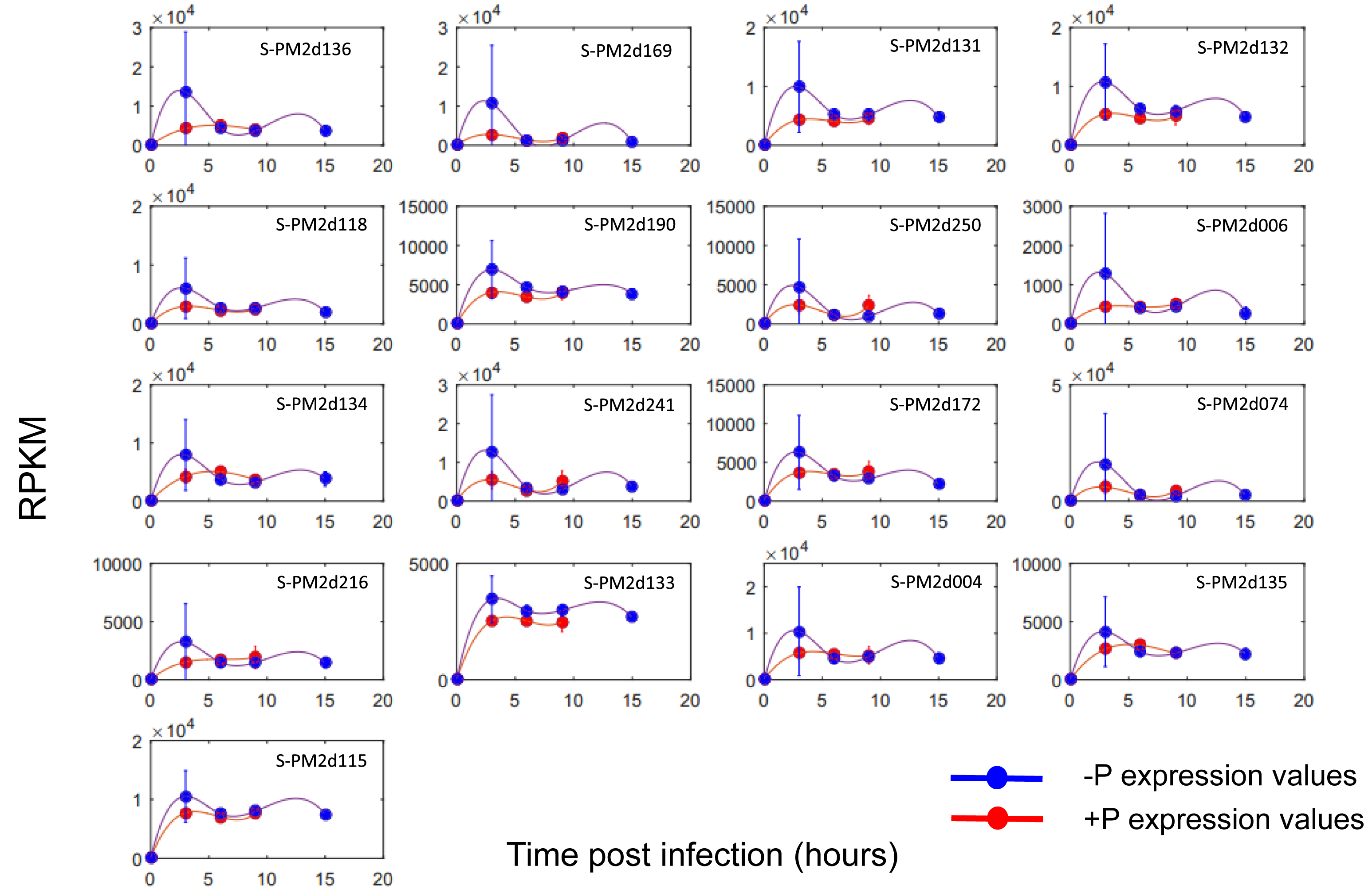


**Supplementary Figure 3:** Electrophoretic Motility Shift Assays (EMSA) of purified MBP-PhoB from *Synechococcus* sp. WH7803 with specific cyanophage S-PM2d gene promoters (S-PM2d130 and S-PM2d136) that did not exhibit binding. The concentration of PhoB protein used ranged from 0-1 µM whilst 25 ng DNA fragment was used in each case. (-): negative control, PhoB with an internal fragment of the *Synechococcus* sp. WH7803 *phoB* gene. (+): positive control, PhoB with the promoter region of the *Synechococcus* sp. WH7803 *phoB* gene.

**
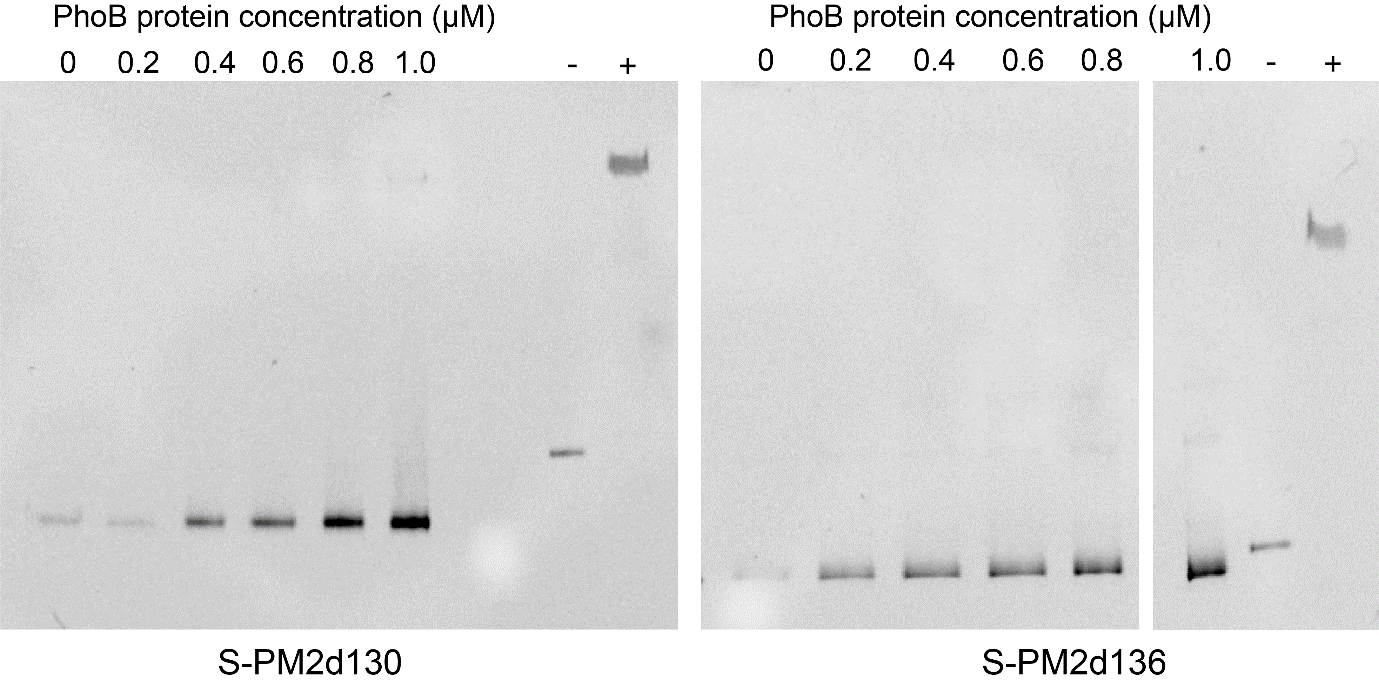
**

**Supplementary Figure 4:** Competitive binding assays of *Synechococcus* sp. WH7803 PhoB to the upstream regions of specific S-PM2d genes. Electrophoretic mobility shift assays of (a) PhoB (1.5 µM) with 20 ng of FAM-labelled S-PM2d134 and 25 ng of S-PM2d004 promoter regions (zero lanes) and then with an increasing concentration of each specific unlabelled fragment with the ratio of labelled:unlabelled fragment noted above the gel. The decreasing intensity of the shifted fragment with increasing ratio of unlabelled fragment demonstrates the specificity of binding. (b) In contrast, PhoB (1.5 µM) bound to 20 ng labelled S-PM2d134 and 25 ng labelled S-PM2d004 promoter regions


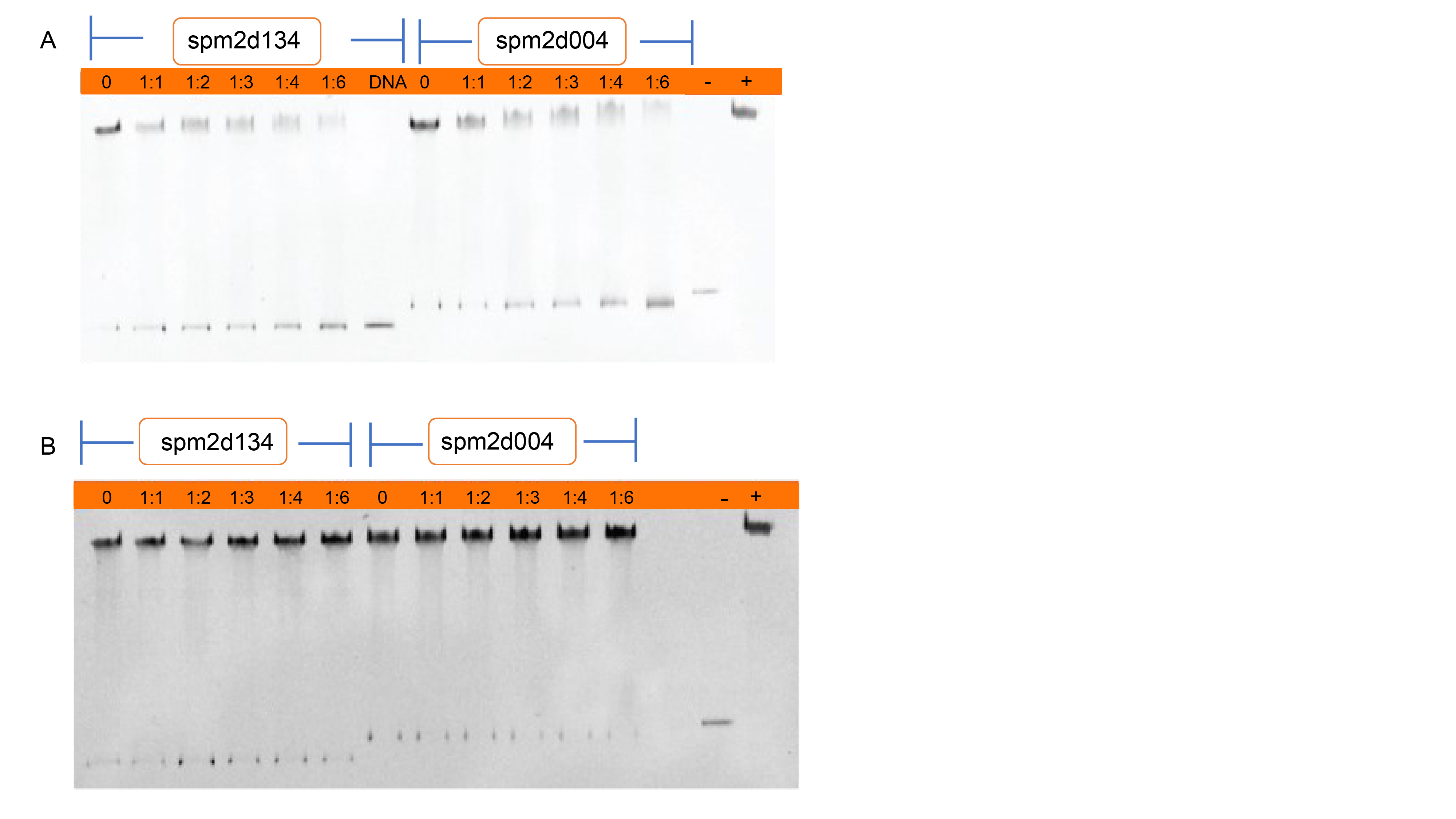


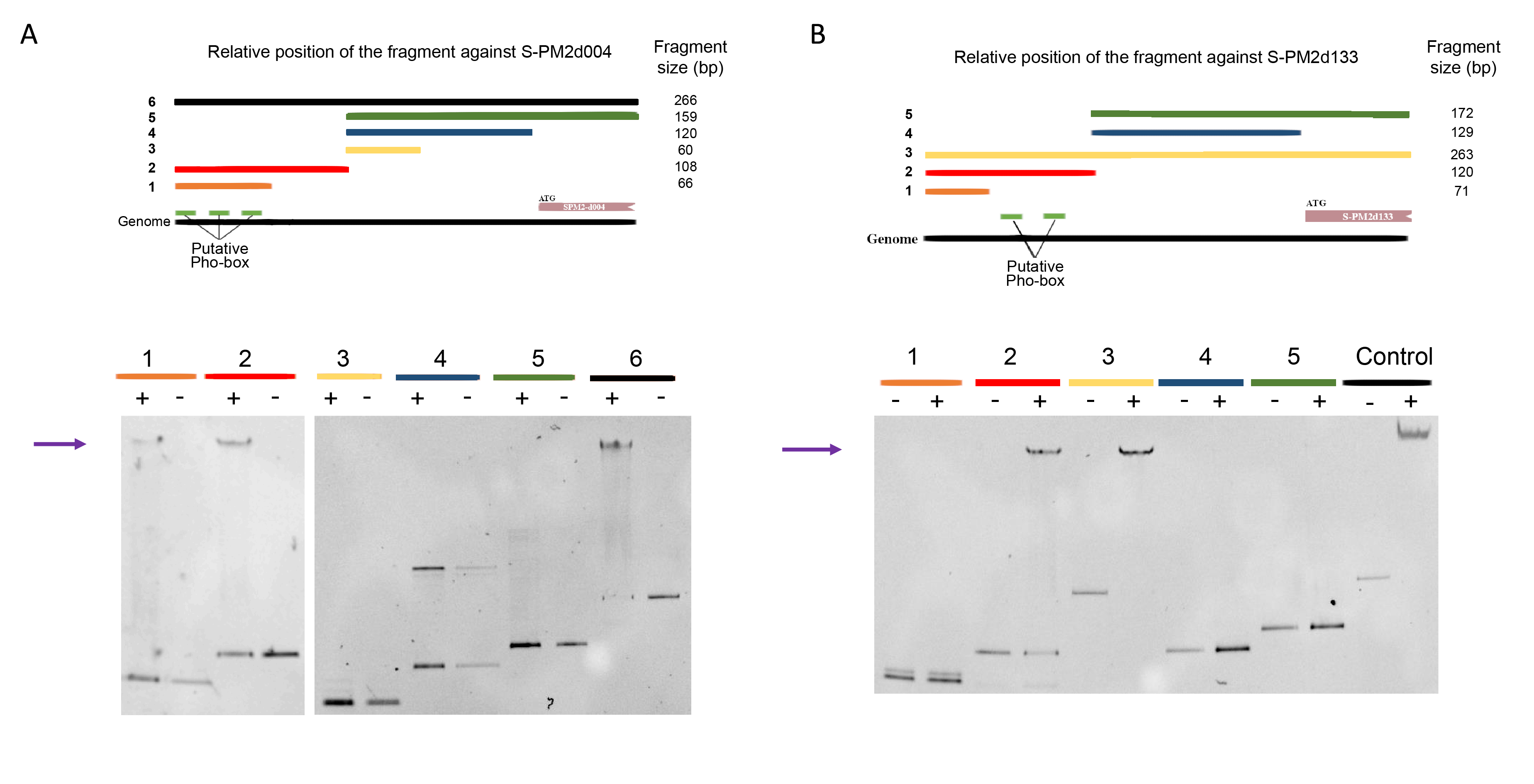
**Supplementary Figure 5:** Electrophoretic mobility shift assays (EMSAs) using the *Synechococcus* sp. WH7803 MBP-PhoB and full-length or truncated upstream promoter regions of the S-PM2d004 gene (A) or S-PM2d133 gene (B) containing, or non-containing, the putative Pho box region. Experiments were performed in the presence (+) or absence (-) of MBP-PhoB protein.

**Supplementary Figure 6:** Electrophoretic Motility Shift Assays (EMSA) of purified MBP protein produced by cleaving the MBP-PhoB fusion protein used in this work. The concentration of MBP protein used ranged from 0-5 µM whilst 25 ng DNA fragment was used in each case. (+): positive control, 1 µM of MBP-PhoB incubated with the promoter region of each gene.


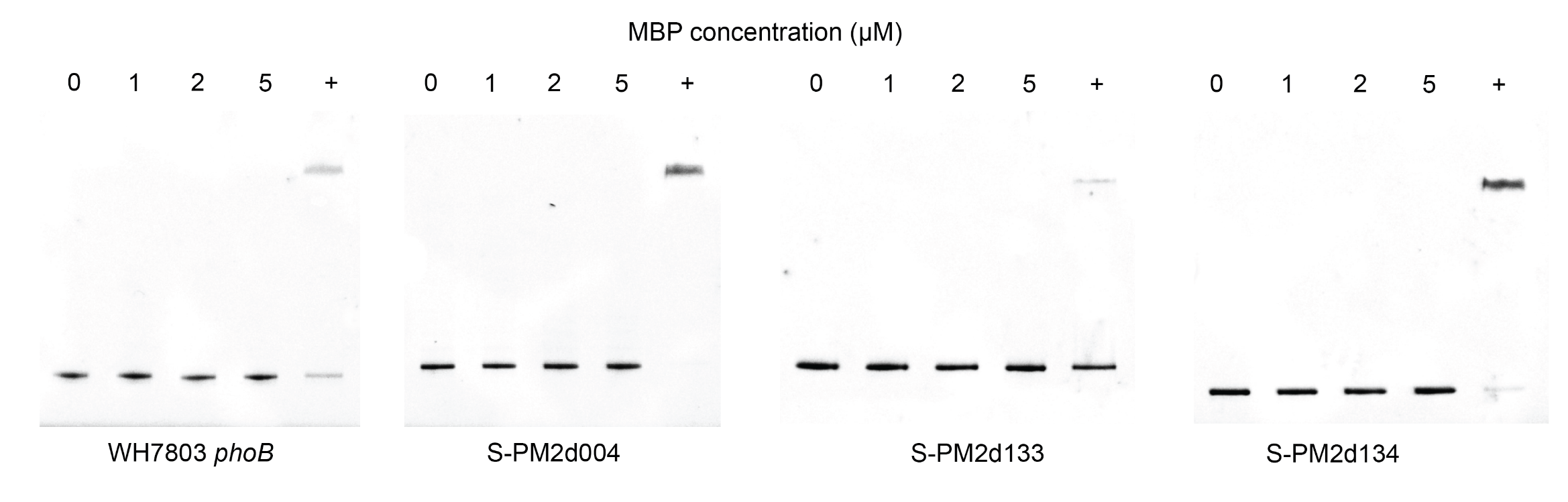


**Supplementary Figure 7:** Phylogenetic tree of cyanophage DNA polymerase sequences. Bootstrap values above 0.8 are noted by the light-blue circles on branch junctions. Bootstrap values >80% are indicated by black circles on branch junctions. Only values >80% are shown. Tree scale represents 1 amino acid substitution. Star symbols next to specific cyanophage strains mark those strains which were used to compare the synteny of genes flanking DNA polymerase in that specific genome. The tree is rooted to the sequence of *E*. *coli* DNA polymerase (Genbank accession number: AAA24407.1)


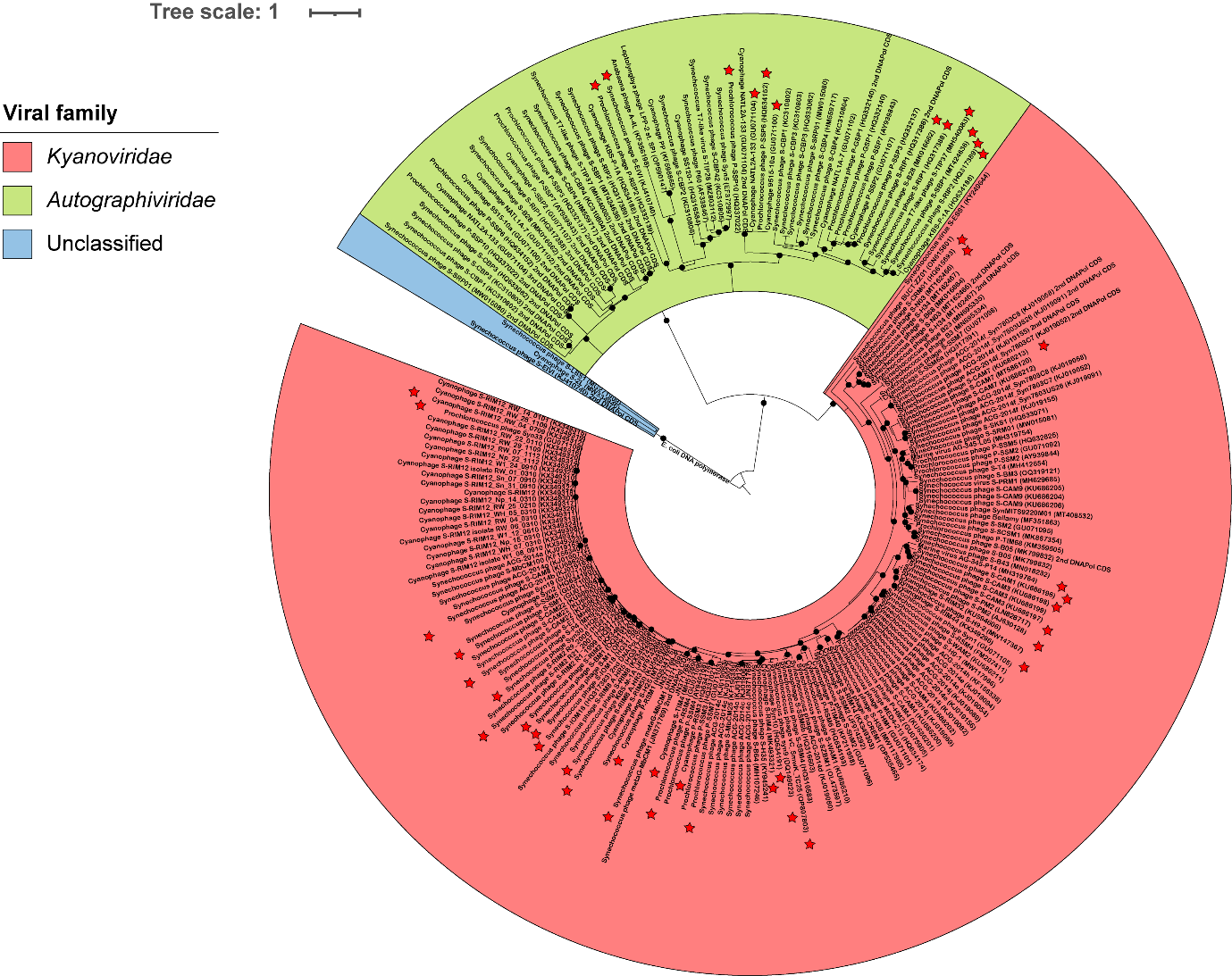


**Supplementary Figure 8:** Genomic context of genes flanking DNA polymerases from different cyanophage genomes belonging to the *Kyanoviridae* (A) and *Autographiviridae* (B) families. Genbank accession numbers of each of the cyanophage genomes is noted under the species name.


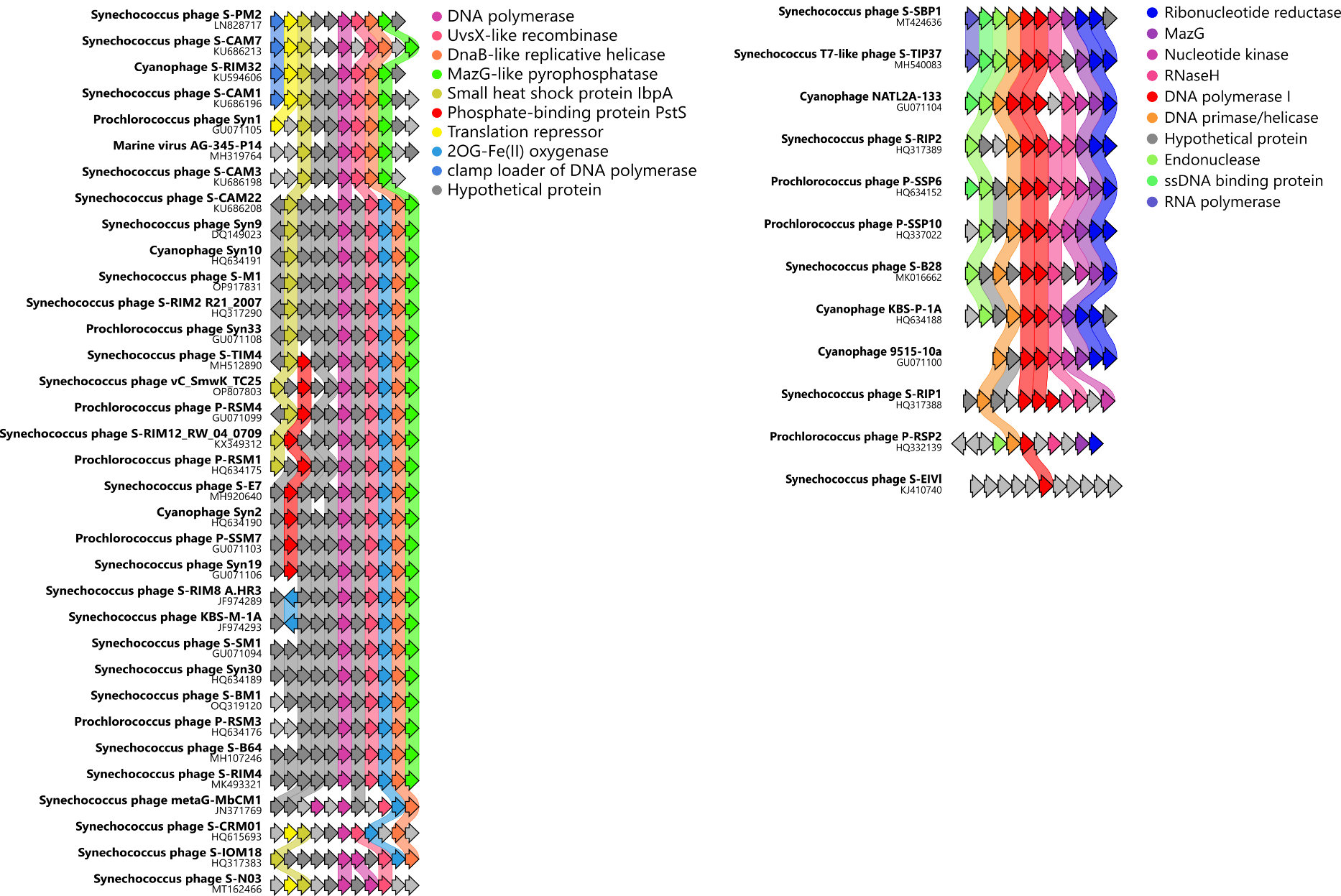


Supplementary Tables

**Supplementary Table 1**: List of PCR primers used in this study.

| **Primer name** | **Primer sequence** | **Primer use** |
| --- | --- | --- |
| SPM2d004_F | 5’- AGTTCTTCCCGAGGATTT -3’ | Primers used to amplify the promoter region of the S-PM2d004 gene |
| SPM2d004_R | 5’- GTGAAGCTGCCGTTATC-3’ |  |
| SPM2d004_-80R | 5’- ACTGGATCGGTTCCCCACATA -3’ | Primers used to amplify different lengths of the promoter region of the S-PM2d004 gene |
| SPM2d004_-100R | 5’- TGGTTAAATGAGAGATAAAGT -3’ |  |
| SPM2d004_-40R | 5’- AGATAACGTTCCAGTTCGGT -3’ |  |
| SPM2d004_100R | 5’- TTTTCTCCAAGAATTGTATA -3’ |  |
| SPM2d004_80F | 5’- TACAGCAAAACTATTTGCAA -3’ |  |
| SPM2d133_F | 5’- AGGGGTACTGGATCATTTCT -3’ | Primers used to amplify the promoter region of the S-PM2d133 gene |
| SPM2d133_R | 5’- TACCTGCTTGTTGTACG -3’ |  |
| SPM2d133_Rev_-100 | 5’- TAACATACAATCGGGGTCCCC -3’ | Primers used to amplify different lengths of the promoter region of the SPM2d133 gene |
| SPM2d133_Rev_80 | 5’- ATGGTGGCCATGGGACGAGG-3’ |  |
| SPM2d133_Forw_80 | 5’- ACAAGGGGTCCTCGTCCCAT -3’ |  |
| SPM2d133_Rev_100 | 5’- TCTGATTTCAATTTGGCATA -3’ |  |
| SPM2d134_F | 5’- CTTGAGAACACCAAACC -3’ | Primers used to amplify the promoter region of the S-PM2d134 gene |
| SPM2d134_R | 5’- TACACTATCTAGGAAACTCA -3’ |  |
| SPM2d136_F | 5’- AGAACAATCCAGTCGTATTATGG -3’ | Primers used to amplify the promoter region of the S-PM2d136 gene |
| SPM2d136_R | 5’- GTAACTTCCGACACAAATTCT -3’ |  |
| SPM2d130_F | 5’- TGAGATCCTAATTCAAACTCGCT -3’ | Primers used to amplify the promoter region of the S-PM2d130 gene |
| SPM2d130_R | 5’- CAGCAAGAGCGACTTCAATTT -3’ |  |
| SPM2d1144_F | 5’- TGAAGGAGGAAACTGAAA -3’ | Primers used to amplify the promoter region of the S-PM2d144 gene |
| SPM2d144_R | 5’- GTCAACGGCAGAATCTA -3’ |  |
| phoB_Promoter_F | 5’- CGATGTCACGCAGGT -3’ | Primers used to amplify the promoter region of the *Synechococcus* sp. WH7803 *phoB* gene |
| phoB_Promoter_R | 5’- ATCATCGTCTTCCACCAC -3’ |  |
| phoB_intra_F | 5’- TGGTGGTGGAAGACGATGAT -3’ | Primers used to amplify the intragenic region of the *Synechococcus* sp. WH7803 *phoB* gene |
| phoB_intra_R | 5’- CCAAAGGGCTTGACCAGATA -3’ |  |
| psbA_SPM2 F | 5’-CTGGTCTGGGTATGGAGGTG-3’ | Primers used for qPCR enumeration of cyanophage S-PM2d in one step infection experiments |
| psbA_SPM2 R | 5’-TGTCGGACGCTTATTCCTGT’’ |  |
| PstS_Deg_F | 5’-TGGTTCAGATCCCGATGACT-3’ | Degenerate primers used to identify the presence of *pstS* in cyanophage isolates |
| PstS_Deg_R | 5’-CACATAGTTRCCTGCRTTGT-3’ |  |

**Supplementary Table 2**: List of genome accession numbers of sequenced marine cyanophages whose *pstS* sequence was used for degenerate *pstS* primer design.

| Cyanophage | Sequence ID |
| --- | --- |
| S-SM1 | GU071094.1 |
| Syn30 | HQ634189.1 |
| S-SM5 | GU071095.1 |
| Syn19 | GU071106.1 |
| S-SKS1 | HQ633071.1 |
| Syn2 | HQ634190.1 |

**Supplementary Table 3**: Cyanophage isolates indicating the isolation location and presence/absence of *pstS* in the context of a delayed lysis phenotype during infection of *Synechococcus* sp. WH7803 under P-deplete conditions.

| Cyanophage | Environment from which isolated | Location of isolation | Method of infection used | | Delayed lysis in low P | Presence of *pstS* (examined using PCR) |
| --- | --- | --- | --- | --- | --- | --- |
|  |  |  | Plaque assay | One-step infection |  |  |
| S-PM2 | Eutrophic | Plymouth Sound, English Channel, UK | **+** | **+** | **+** | **-** |
| S-WHM1 | Eutrophic | Woods Hole Oceanographic Institute harbour, Woods Hole, Massachusetts, USA | **+** | **+** | **+** | **-** |
| Syn9 | Eutrophic |  | **+** | **+** | **+** | **-** |
| S-RSM60 | Oligotrophic | Gulf of Aqaba, Red Sea | **+** | **+** | **+** | **-** |
| S-RSM61 | Oligotrophic |  | **-** | **+** | **+** | **-** |
| S-RSM62 | Oligotrophic |  | **-** | **+** | **+** | **-** |
| S-RSM63 | Oligotrophic |  | **-** | **+** | **+** | **-** |
| S-RSM64 | Oligotrophic |  | **-** | **+** | **+** | **-** |
| S-RSM65 | Oligotrophic |  | **-** | **+** | **+** | **-** |
| S-RSM66 | Oligotrophic |  | **-** | **+** | **+** | **-** |
| S-RSM67 | Oligotrophic |  | **-** | **+** | **+** | **-** |
| S-RSM68 | Oligotrophic |  | **-** | **+** | **+** | **-** |
| S-RSM69 | Oligotrophic |  | **-** | **+** | **+** | **-** |
| S-RSM70 | Oligotrophic |  | **-** | **+** | **+** | **-** |
| S-RSM71 | Oligotrophic |  | **-** | **+** | **+** | **-** |
| S-RSM72 | Oligotrophic |  | **-** | **+** | **+** | **-** |
| S-BM1 | Oligotrophic | Coastal waters of the Sargasso Sea | **+** | **+** | **-** | **+** |
| S-BM3 | Oligotrophic |  | **-** | **+** | **+** | **-** |

**Supplementary Table 4**: RNA-Seq read and mapping statistics of *Synechococcus* sp. WH7803 grown under P-replete and P-deplete conditions and in the presence and absence of infection by cyanophage S-PM2d. Fold coverage is calculated as the number of reads multiplied by the read length (15bp) and divided by the genome size. NS – no sample was taken for this time point and condition.

|  |  |  | Aligned reads | | | Computed fold coverage | |
| --- | --- | --- | --- | --- | --- | --- | --- |
| Sample | | Total reads | S-PM2d | WH7803 (total) | rRNA | S-PM2d | WH7803 |
| +P uninfected | T0 | 45,835,981 | 90,667 (0.20%) | 39,044,970 (85.18%( | 294,458 (0.64%) | 72.83 | 2,474.35 |
|  | T3 | 42,955,097 | 92,998 (0.22%) | 37,044,630 (86.24%) | 176,731 (0.41%) | 74.70 | 2,347.59 |
|  | T6 | 45,437,628 | 376,272 (0.83%) | 41,267,449 (90.82%) | 138,930 (0.31%( | 302.25 | 2,615.20 |
|  | T9 | 46,563,971 | 864,069 (1.86%) | 41,856,958 (89.89%) | 208,411 )0.45%( | 694.08 | 2,652.55 |
|  | T12 | 46,578,331 | 315,017 (0.68%) | 41,378,285 (88.84%) | 269,553 (0.58%) | 253.04 | 2,622.22 |
|  | T15 | 57,689,177 | 191,652 (0.33%( | 53,064,767 (91.98%) | 282,354 (0.49%) | 153.95 | 3,362.81 |
| +P infected | T0 | NS | NS | NS | NS | NS | NS |
|  | T3 | 64,708,906 | 62,589,646 (96.72%) | 1,094,275 (1.69%) | 14,548 (0.02%) | 50,276.58 | 69.35 |
|  | T6 | 45,611,926 | 44,773,750 (98.16%) | 417,687 (0.92%) | 5,854 (0.01%( | 35,965.55 | 26.47 |
|  | T9 | 61,605,022 | 59,001,150 (95.77%( | 1,043,947 (1.69%( | 25,903 (0.04%) | 47,394.03 | 66.16 |
| -P uninfected | T0 | 51,571,921 | 90,077 (0.17%( | 47,883,066 (92.85%) | 290,222 (0.56%) | 72.36 | 3,034.44 |
|  | T3 | 52,868,975 | 204,113 )0.39%( | 48,935,389 (92.56%) | 202,218 (0.38%) | 163.96 | 3,101.13 |
|  | T6 | 56,975,818 | 703,449 (1.23%) | 52,139,206 (91.51%) | 219,097 (0.38%( | 565.06 | 3,304.16 |
|  | T9 | 49,340,614 | 1,785,182 (3.62%( | 42,690,551 (86.52%) | 217,053 (0.44%) | 1,433.99 | 2,705.38 |
|  | T12 | 51,479,973 | 850,823 (1.65%) | 46,528,201 (90.38%) | 215,280 (0.42%) | 683.44 | 2,948.58 |
|  | T15 | 57,485,017 | 366,150 (0.64%) | 52,698,190 (91.67%) | 334,278 (0.58%) | 294.12 | 3,339.58 |
| -P infected | T0 | NS | NS | NS | NS | NS | NS |
|  | T3 | 51,162,704 | 45,515,772 (88.96%) | 2,257,859 (4.41%) | 64,086 (0.13%) | 36,561.59 | 143.08 |
|  | T6 | 59,600,741 | 56,204,379 (94.30%) | 1,647,048 (2.76%( | 27,496 (0.05%) | 45,147.46 | 104.38 |
|  | T9 | 56,776,022 | 53,626,948 (94.45%) | 1,231,052 (2.17%) | 25,571 (0.05%( | 43,077.08 | 78.01 |
|  | T12 | 64,304,977 | 59,837,063 (93.05%) | 1,344,363 (2.09%) | 81,317 (0.13%) | 48,065.50 | 85.19 |
|  | 15 | 53,647,162 | 49,583,494 (92.43%) | 1,255,848 (2.34%) | 44,638 (0.08%) | 39,829.09 | 79.59 |

**Supplementary Table 5**: List of *Kyanoviridae* core genes used to generate the cyanophage core genome tree shown in Figure 1A.

| **Gene** | **Annotation** |
| --- | --- |
| dexA | 3'-5' exonuclease |
| thyA | Thymidylate synthase |
| T4_GP13 | Neck protein |
| T4_GP14 | Head-to-tail connector protein |
| T4_GP15 | Tail terminator protein |
| T4_GP16 | DNA packaging protein subunit |
| T4_GP17 | DNA packaging protein subunit |
| T4_GP18 | Tail sheath protein |
| T4_GP19 | Tail tube protein |
| T4_GP21 | Procapsid protease |
| T4_GP22 | Prohead scaffolding core protein |
| T4_GP23 | Major capsid protein |
| T4_GP25 | Sheath polymerisation initiator |
| T4_GP26 | Baseplate protein |
| T4_GP32 | ssDNA binding protein |
| T4_GP33 | Late transcription coactivator |
| T4_GP4 | Head packaging nuclease |
| T4_GP41 | DNA helicase |
| T4_GP43 | DNA polymerase |
| T4_GP44 | Clamp loader protein subunit |
| T4_GP45 | Sliding clamp protein |
| T4_GP46 | Recombination exonuclease subunit |
| T4_GP47 | Recombination exonuclease subunit |
| T4_GP48 | Baseplate tube cap protein |
| T4_GP5 | Spike protein |
| T4_GP53 | Baseplate protein |
| T4_GP55 | Late transcription coactivator |
| T4_GP6 | Tail sheath protein |
| T4_GP61 | Primase |
| T4_GP62 | Clamp loader protein subunit |
| T4_GP8 | Baseplate structural protein |
| T4_nrdA | Ribonucleotide reductase |
| T4_nrdB | Ribonucleotide reductase |
| T4_nrdC | Glutaredoxin |
| T4_regA | Translation regulatory protein |
| T4_uvsX | DNA recombinase |
| T4_GC_142 | Hypothetical protein |
| T4_GC_146 | Hypothetical protein |
| T4_GC_15 | Hypothetical protein |
| T4_GC_150 | Hypothetical protein |
| T4_GC_152 | Hypothetical protein |
| T4_GC_170 | Hypothetical protein |
| T4_GC_176 | Hypothetical protein |
| T4_GC_184 | Hypothetical protein |
| T4_GC_190 | Hypothetical protein |
| T4_GC_198 | Hypothetical protein |
| T4_GC_201 | Hypothetical protein |
| T4_GC_250 | Hypothetical protein |
| T4_GC_267 | Hypothetical protein |
| T4_GC_280 | Hypothetical protein |
| T4_GC_313 | Hypothetical protein |
| T4_GC_321 | Hypothetical protein |
| T4_GC_322 | Hypothetical protein |
| T4_GC_4 | Hypothetical protein |
| T4_GC_43 | Hypothetical protein |
| T4_GC_49 | Hypothetical protein |
| T4_GC_71 | Hypothetical protein |
